# Spore-Forming Clostridia in Raw Cow Milk from Northern Italy: A Trend Analysis over the Past 20 Years

**DOI:** 10.3390/foods13223638

**Published:** 2024-11-14

**Authors:** Arianna Guaita, Lorenzo Gambi, Pierluigi Baresi, Franco Paterlini, Giuseppe Bolzoni, Giorgio Zanardi, Paolo Daminelli

**Affiliations:** 1 National Reference Center for Bovine Milk Quality, Via A. Bianchi 9, 25124 Brescia, Italy; paolo.daminelli@izsler.it; 2 “Produzione Primaria” Department, Istituto Zooprofilattico Sperimentale della Lombardia e dell’Emilia Romagna “Bruno Ubertini” (IZSLER), Via A. Bianchi 9, 25124 Brescia, Italyfranco.paterlini@izsler.it (F.P.);

**Keywords:** *Clostridium* spp., spores, cheese spoilage, semi-quantitative MPN, annual and seasonal trend

## Abstract

*Clostridium* species are known for their impact on animal and human health, but also for the spoilage of foodstuffs. Their spores contaminate milk and result in germination and gas production, the latter being particularly evident in the cheeses that suffer severe depreciation. To address this issue, the Primary Production Department of the IZSLER institute in Brescia, Italy conducts the Most Probable Number (MPN) method on bovine milk samples collected from Northern Italian dairies between 2004 and 2023. This approach leverages two semi-quantitative protocols, S2 and S3, to detect *Clostridium* species spore forms upon customer request. Here, we would like to present an a-posteriori analysis on the results of the S2 and S3 protocols. The goal of this study is to highlight the differences between these two methods and provide evidence of the actual decrease in *Clostridium* species in raw cow milk over a 20-year period. Our analysis shows that client demand for S2 has progressively decreased, while S3’s has remained constant, and both protocols reveal a significant reduction in positives; furthermore, S3’s greater sensitivity made it more responsive to environmental changes. This highlights the necessity of choosing the appropriate testing protocol that accounts for both regulatory standards and environmental factors. Overall, our findings underscore the importance of continued monitoring to manage *Clostridium* species contamination and ensure milk quality.

## 1. Introduction

The genus of *Clostridium* consists of gram-positive spore-forming anaerobic bacteria responsible for quality defects in food and feed, especially in hard and semi-hard cheese such as Grana Padano PDO (Protected Designation of Origin), Gouda, Emmental, and Gruyere [1]. The main species involved in cheese defects are *Clostridium tyrobutyricum*, *Clostridium butyricum*, *Clostridium sporogenes*, and *Clostridium beijerinckii*, all of which are included in the Butyric Acid Bacteria group (BABs), as they produce butyric acid. In particular, *C. tyrobutyricum* and *C. beijerinckii* are the main gas producers; hence, they are primarily responsible for late defects [2].

BABs convert lactate into gases (H_2_ and CO_2_), acetic acid, and butyric acid causing Late Blowing Defects (LBDs), cracks, cavities called “eyes”, shredding, and openings in the central part of the form, changes in flavor, and a spongy consistency that decrease the commercial value of cheese [3,4]. All these defects can occur at a very low concentration of spores, approximately 100 to 1000 clostridial spores per liter of milk [5,6]. This is why it is critical to pay close attention to clostridial milk contamination from a variety of sources, including crops, bedding, bulk tanks, feces, poor handling, contaminated equipment, and soils [7,8].

*Clostridium* spores are a major problem, as they are resistant to various cheese-manufacturing procedures. For example, pasteurization kills most vegetative bacterial cells, but not clostridial spores, which remain quiescent until germination [9,10]. A few weeks into ripening, the physical and chemical conditions of cheese become optimal for reactivation of the clostridial spores, leading to LBDs. Reducing the spore concentration in milk is possible through outcropping—a natural stage in some cheese production—or other methods such as bactofugation, microfiltration, or the addition of bacterial strains that compete with *Clostridium* for lactic acid metabolism [11]. The use of preservatives or adjuvant technology like nitrates and lysozyme (500 U/mL cheese milk) is also effective, especially if the clostridial spore concentration is less than 300 per liter [12,13]. However, these substances are permitted only under specific productions (e.g., up to 2.5 g of lysozyme per 100 kg of milk in Grana Padano PDO production [14]) or are completely forbidden (e.g., in Parmigiano Reggiano PDO production [15]).

In 2015, 15–35% of Grana Padano PDO production displayed non-compliant forms that presented defects partially caused by *Clostridium* [16]. In 2022, these defects decreased to 2% [17,18,19], but still led to an economic loss of EUR ~36 million [20]. This highlights the importance of detecting clostridial spores in milk samples for a prediction of the ripening outcomes. In 2016, Brändle at al. proposed a classification of the available laboratory methods to quantify the spore levels in milk [21]. Most of these approaches are culture-dependent and rely on plate counting or a Multiplex-PCR to distinguish the different *Clostridium* species present in the samples and ensure a precise quantification [4,11]. However, the high costs and small number of samples that can be analyzed in a day pose significant challenges [16,22]. The rapid, automated methods for spore detection that are commercially available (e.g., AMP6000) have a higher selectivity medium, but often require expensive equipment that most laboratories cannot afford [23,24].

The Most Probable Number (MPN) method on a liquid medium is currently the most widely used method for assessing the contamination of cow’s milk by sporulating forms of *Clostridium* spp. The process involves seeding the milk sample into tubes, pasteurizing either before or after the seeding, and adding a paraffin plug. If the spores in the sample germinate and produce CO_2_, the plug will detach from the surface, indicating contamination [21]. Notably, the MPN method cannot differentiate between germinated *Clostridium* species or determine their relevance to food spoilage.

In the Primary Production Department of the IZSLER institute in Brescia, Italy, the MPN method, as reported in the International Organization for Standardization (ISO) 7218:2013 [25], is employed to semi-quantitatively assess clostridial contamination in milk. This method is a commercial parameter within the Milk Quality Payment framework [26,27], determining milk premiums or penalties based on quality. It also adheres to Regulation (EC) No 853/2004 of the European Parliament and Council, which sets specific hygiene rules for food of animal origin, including dairy products [28].

The purpose of this study is to analyze the Milk Quality Payment data collected by IZSLER’s Primary Production Department and the National Reference Centre for Cow Milk Quality for the years 2004–2023 applying two MPN protocols, S2 and S3. Although conceptually similar, these two protocols differ in sensitivity according to the number of dilutions performed on the bovine samples: two for S2 and three for S3. In addition, the S2 protocol is used as a general screening test, whereas the S3 protocol is mainly used on milk intended for cheesemaking, which therefore needs to follow more accurate controls regarding sporal contamination. Here, we provide an overview of the trend of *Clostridium* species in the field samples submitted to the institute by breeders and dairies in Northern Italy. Since more than 40% of lactating cows with dirty udders can increase the average contamination of the spores in milk by 15% [29,30,31], evaluating these trends helps with understanding how the evolution of farm practices impacts the presence of sporigenic bacteria in milk. Even though BABs bacteria causing late defects in dairy products do not pose a risk to consumers [21,32], the *Clostridium* genus remains potentially toxic, neuro-toxigenic in addition to food spoiling [33]. Our 20-year review of the S2 and S3 analyses for the detection of *Clostridium* species shows that the constant application of the MPN method has helped control the spread of this bacterial genus [34].

## 2. Material and Methods

### 2.1. Sample Collection

As the alterations induced by LBDs affect the quality of long-ripened cheeses made from raw cow milk, we investigated the hygienic and commercial parameters of the raw milk samples destined for cheese-making. All the raw milk samples analyzed and reported in this study were taken by samplers, delivered to IZSLER by private companies in a refrigerated state, processed on the day of arrival, and then discarded, as their shelf-life is 3 days.

The samples, or matrices, included in the analysis were cow milk, boiler milk, raw mass milk, and unpasteurized bovine whole milk. In contrast, goat milk, goat mass milk, sheep milk, buffalo milk, mare milk, bovine whey, pasteurized bovine milk, Ultra-High Temperature (UHT) bovine milk, and cream were excluded (Table 1).

Over 95% of the raw milk samples originated from Northern Italian provinces, specifically Lombardy and Emilia Romagna, particularly in the Grana Padano PDO area (Table 2). Consequently, our analysis provides insights on the contamination levels and regional prevalence of *Clostridium* species within this specific geographical region.

### 2.2. MPN Protocols

The MPN method presented here was performed according to two analogous protocols, S2 and S3, based on customer request. Both protocols analyze raw bovine milk samples diluted at 1:10 and 1:100 ratios; to these two dilutions in the S3 protocol a third analytical step is added where undiluted samples are analyzed.

The raw milk is diluted with a BUtyricum and TIrobutyricum (BUTI) medium, which consists of 0.1% casein tryptic digest, 0.1% meat extract, 0.03% yeast extract, 0.08% sodium acetate, 0.05% sodium chloride, 0.01% soluble starch, 25 mL of 50% sodium lactate, and 975 mL of demineralized water. In addition, in the S3 application of the MPN method, 0.3 mL of 50% sodium lactate, which promotes the germination of *Clostridium* spp. spores, must be added. Completely dissolve the reagents in demineralized water preheated to 50 °C; adjust the pH to 6.1 (37% hydrochloric acid is often needed) and sterilize the solution by autoclaving at 121 °C for 15 min. Store it at 4 °C for up to 30 days, away from direct light to prevent photo-oxidation.

A total of 10 mL of raw milk (undiluted, 1:10, 1:100) are seeded in sterile 16 mL × 160 mm glass tubes containing 2 mL of a paraffin and Vaseline oil mixture (3:1 ratio). For the S3 undiluted samples, 0.3 mL in 50% solution sodium lactate (C_3_H_5_NaO_3_) is directly added to the glass tube.

After seeding the medium, the tubes are pasteurized at 85 °C for 20 min and cooled at 15 °C for 10 min. This process causes the paraffin and Vaseline oil mixture to melt, then solidify, forming a non-permeable gas plug that seals the sample in a tight anaerobic environment. Following incubation at 37 °C for 7 days, the tubes are checked individually to assess whether the oil plug had detached from the underlying medium due to CO_2_ and H_2_ production, indicating the presence of germinated spores in the sample.

The MPN method explained here [25,27,35,36], can be performed manually or with automatic sample preparers such as the ones employed by the company Skalar; in our case, the analyses were carried out using automatic procedures [37]. Each protocol is performed in triplicate. For every 60 samples analyzed, a control is analyzed to verify that the machine is not affected by entrainment and that the medium in use is not contaminated. The standard consists of sterile distilled water, added in the same dilution as the analyzed milk sample.

### 2.3. MPN Validation

The MPN values were calculated with the Thomas formula [38]:

(1) $$MPN=\frac{P}{\sqrt{n\times T}}$$

where P is the number of positives in the set of seeded tubes, n is the volume of the inoculated sample (L), and T is the total volume of the reaction tube (L). The MPN method presented here was validated for accuracy, precision, specificity, and the Limit of Detection (LOD) as required by ISO 9001:2000, in force at the time of validation and recently updated to 17025:2018 [39]. The uncertainty of the estimated MPN value measured was expressed through the fiducial limits with a 95% confidence level (P = 95%, K ≈ 2) in the S2 protocol, as specified in ISO 7218:2007 Amd 1:2013 [25]. In contrast, for S3, the standards do not specify explicit calculation methods. Therefore, we determined the uncertainty as the sum of the relative uncertainties associated with dilution and the MPN estimation, as described in the bibliography [38]. To assess the robustness of the method, several factors were taken into account for their influence on the analytical result such as the incubation time.

### 2.4. Data Analysis

The sensitivity of the MPN method was set at 308 spores/L for the S2 protocol and 31 spores/L for the S3 protocol. Both methods have an upper detection limit of 11,000 spores/L, as required by ISO 7218:2007 Amd 1:2013 [25]. S3 has higher sensitivity due to the inclusion of an undiluted sample, enabling the detection of lower spore concentrations.

To make the classification of the positive samples more interpretable, the results were divided into weak (Pos) and strong (Pos+) positives based on the spore count ranges. For the S2 protocol, the samples within the 308–966 spores/L range were classified as Pos, while those with more than 967 spores/L were classified as Pos+. For the S3 protocol, the samples with 31–200 spores/L were considered Pos, and those with 201 spores/L or more were Pos+. This approach allowed us to capture meaningful variations without introducing unnecessary complexity.

The data were analyzed using one-way ANOVA (α = 0.01) on monthly frequencies, with post-hoc comparisons made using the Tukey–Kramer test (α = 0.01) to compare each year’s relative frequency with the 2004 average. The Shapiro–Wilk test (α = 0.01) was applied to check for normality within each year and category. The outliers were excluded using the Z statistic. All the analyses were performed using the Microsoft Excel statistical package.

## 3. Results

### 3.1. The Demand for S2 Analysis Declines as S3 Requests Increase

**Figure 1:**
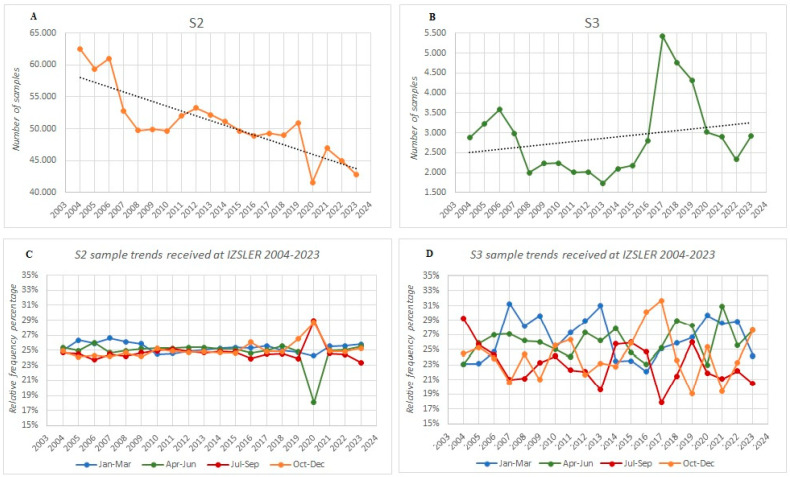
The 20-year trends in the number of samples delivered to and analyzed at IZSLER. Panels (**A**,**B**) display the annual demand for the S2 and S3 protocols, respectively. Panels (**C**,**D**) illustrate the seasonal demand for the same protocols.

We applied the MPN method, specifically the S2 and S3 protocols, to assess spore presence and germination in the raw milk samples from the Northern Italian dairies delivered to IZSLER. Between 2004 and 2023, a total of 1,017,606 raw milk samples were analyzed with the S2 protocol, and 57,575 with the S3 protocol, averaging approximately 50,000 and 3000 samples per year, respectively (Figure 1A,B). The customer demand for an S2 analysis began declining in 2007 and had decreased by 31.4% by 2023. Meanwhile, the S3 requests remained steady, except for a notable increase between 2017 and 2019.

Grouping the data by season, no significant variations were observed in the samples delivered for an S2 analysis (Figure 1C). However, the demand for the S3 protocol showed wider fluctuations across the different seasons (Figure 1D).

### 3.2. Samples Positive for Clostridium Contamination Decreased in the Past 20 Years

By monitoring gas production and paraffin–Vaseline cap detachment in the tubes, we recorded the number of positive results at each dilution, along with the corresponding MPN values and the associated 95% confidence limits (Table 3).

**Figure 2:**
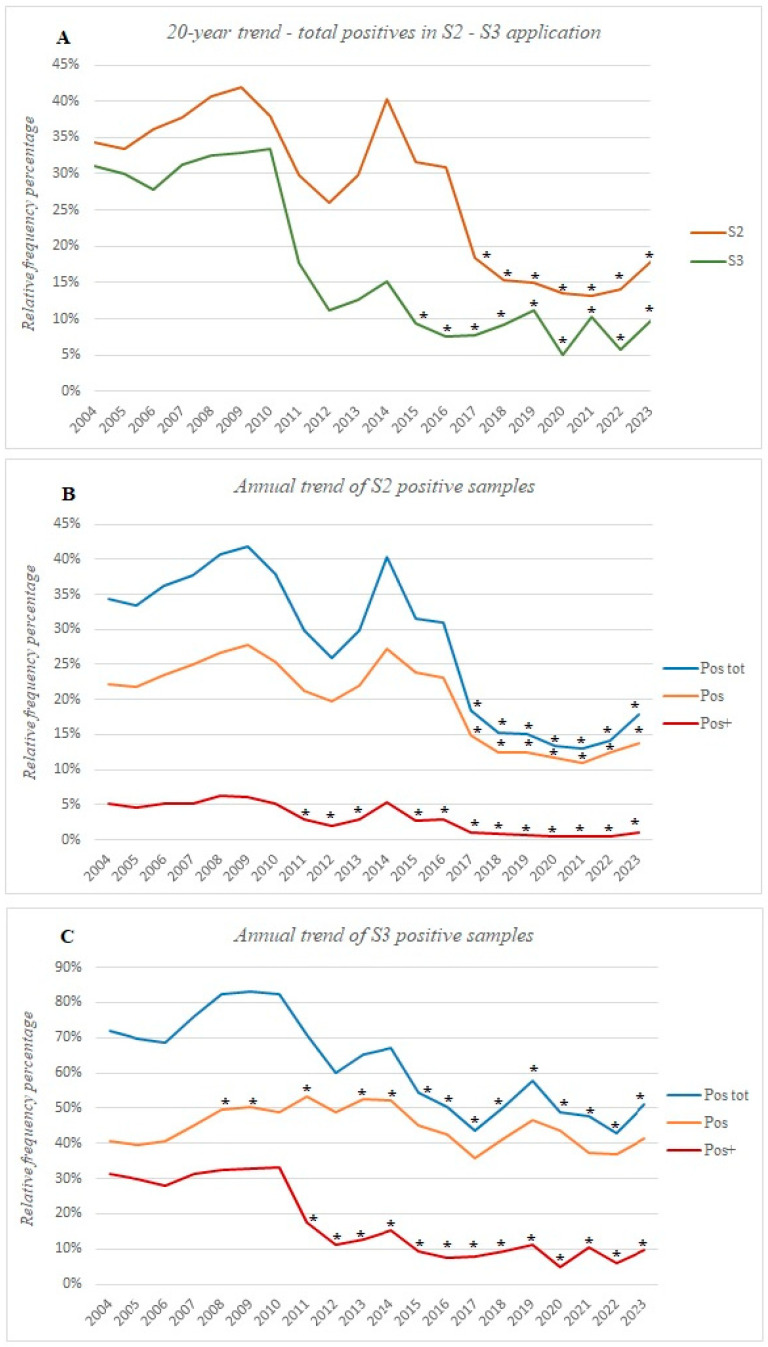
The 20-year annual trend in positive samples from S2 and S3 analyses. Panel (**A**) compares total positive samples (Pos tot) for both protocols. Panels (**B**,**C**) show trends in weak (Pos) and strong (Pos+) positive samples identified with the S2 and S3, respectively. Asterisks (*) denote years with significant differences from 2004 in the relative frequency of positive samples.

Following this, we compared the average percentages of the total positive samples (Pos tot) each year with those from 2004 to identify any significant differences (Figure 2A). This revealed that when using the S2 protocol, the Pos tot decreased by ~16% starting in 2017, while with the S3 protocol the reduction was ~17% starting in 2015. Then, we separated the weak positive samples (Pos) from the strong positives (Pos+) for both S2 and S3, and carried out the same analysis (Figure 2B,C).

Since 2004, the S2 protocol has shown a decrease of ~8% in the number of Pos and ~4% in the number of Pos+, with the first significant peaks observed in 2017 and 2011, respectively. In contrast, the S3 protocol revealed a decrease of about 21% in the number of Pos+, with an increase in the number of Pos in 2008 and 2011. Further information can be found in the Supplementary Materials.

**Figure 3:**
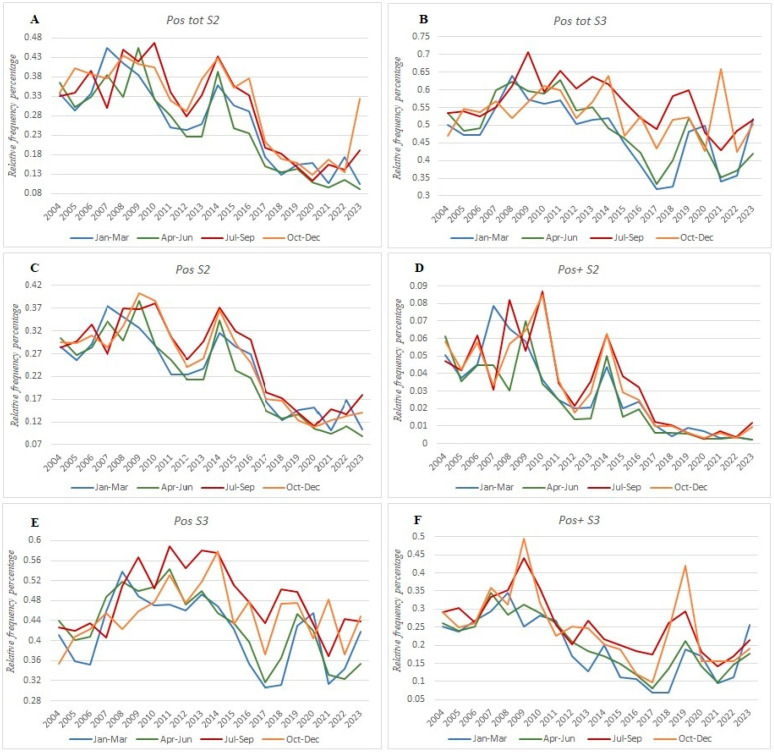
Seasonal distribution of positive samples using the S2 and S3 protocols. Trends for total (Pos tot), weak (Pos), and strong (Pos+) positive samples across seasons are displayed in the panels (**A**,**C**,**D**) for the S2 protocol and (**B**,**E**,**F**) for the S3 protocol, respectively.

Finally, we examined the seasonal variations in the Pos tot, Pos, and Pos+ results. The S2 protocol showed no significant differences across the seasons (Figure 3A). However, the more sensitive S3 protocol revealed a significant increase in the Pos tot from July to September, except for in 2021, where the highest values occurred in October–December (Figure 3B). When breaking down the Pos tot into Pos and Pos+, similar patterns emerged. S2 detected no significant seasonal variation (Figure 3C,D). In contrast, S3 showed an increase in Pos from July to September, though this trend was not sustained by the Pos+, with the exception of October–December of 2019 (Figure 3E,F).

## 4. Discussion

The *Clostridium* genus consists of gram-positive spore-forming anaerobic bacteria that are responsible for quality defects in hard and semi-hard cheese [1,3,4,7]. Thus, the detection of the presence and concentration of *Clostridium* contamination in milk is crucial to prevent economic losses and safeguard public health [33]. In the Quality Milk Payment framework [26] in Italy, the commercial milk limit for clostridial spores is set at 100 spores/L to ensure product quality and safety [5,40]. Since a spore count cannot be measured directly, the institute identifies butyric *Clostridium* in milk by applying either the S2 or S3 protocols of the Most Probable Number (MPN) method [4,16,41] to quantify the spores per liter of milk samples.

### 4.1. S2 and S3 Demand Is Tied to Regulatory Requirements, Dairy Practices, and External Events

In this study, we reviewed 20 years of data on raw milk samples delivered to IZSLER and analyzed them using the S2 and S3 protocols. We examined the sample sizes over the years as well as the customer preferences for these protocols, and we identified the trends in *Clostridium* contamination. This analysis provides insight into how clostridial spore levels have evolved over time as well as how customer demand for testing protocols has shifted, and highlights the patterns related to annual and seasonal variations.

The first trend identified over the past 20 years lies in the customer demand for the S2 and S3 protocols. Our data show a significantly higher number of requests for the S2 protocol compared to S3 (~50,000 vs. 3000 samples per year, Figure 1A,B). This is largely explained by the fact that S2 is a mandatory screening parameter of the Quality Milk Payment Regulation [28], with all milk producers being required to take and analyze at least two samples per month. In contrast, S3 is not compulsory, but it is more sensitive and is thus preferred by the dairies producing medium-long ripening cheese and those using silage-fed cows.

Secondly, we observed a steady decline in S2 demand, while the requests for the S3 protocol remained constant, except for a brief dip between 2017 and 2019 (Figure 1A,B). These trends align with the broader shifts in dairy farming practices. Since 2010, there has been a gradual reduction in dairy cow herds, with smaller farms being replaced by larger ones that produce more milk, often destined for cheese-making [42,43]. This change in farming practices has influenced both the number of milk samples delivered for analysis and the type of tests requested, leading to a decrease in S2 demand and an increase in the demand for the more sensitive S3 protocol.

Comparing the seasonal demands for the protocols, the S2 requests remained constant across the seasons, while the S3 requests exhibited much greater variability (Figure 1C,D). This difference is mainly due to the distinct nature of the two methods. The S2 protocol, being mandatory under the Quality Milk Payment Regulation, is consistently requested and routinely performed. In contrast, S3 is an optional protocol used by diaries at their discretion, often depending on the specific needs related to cheese-making or other factors.

Interestingly, our analysis revealed an anomaly in the number of milk samples analyzed with the S2 protocol during 2020, which can be attributed to the disruptions caused by the COVID-19 pandemic. Specifically, in March and April of 2020, the regions of Lombardy and Emilia Romagna granted temporary exemptions for the analysis of milk samples as part of the Quality Milk Payment regulations. This exemption led to a sharp decrease in the number of S2 protocol samples analyzed during this period (Figure 1A,C). In contrast, the S3 protocol, which was not subject to regulatory requirements, did not show any fluctuations in sample numbers during this time (Figure 1B,D). This indicates that while the pandemic and subsequent regulatory changes impacted the S2 protocol, the demand for the S3 protocol remained constant.

### 4.2. Regulatory Changes and Seasonal Variation Impact on Clostridium-Positive Milk Samples

Our data show a reduction in the total positive samples (Pos tot) observed in both the S2 and S3 protocols (Figure 2A), likely reflecting the impact of the stricter animal welfare and hygiene regulations introduced from 2017 to 2018 [44,45,46]. These measures, such as mandatory udder cleaning, have been proven to reduce clostridia spore transfer in raw milk [47,48], despite the fact that many factors may affect together or independently the ripening process of hard and semi-hard cheese, such as dairy technology, autochthonous starters, and additives. Despite the similar behavior of the two protocols, the results diverged when comparing the total (Pos tot), weak (Pos), and strong (Pos+) positives. S2 detected fewer contaminated samples for both the Pos and Pos+ (~8% and ~4%, Figure 2B), while S3 recorded a more pronounced drop in total positives, mainly due to the reduction in the number of Pos+ (21%, Figure 2C), and an almost unchanged trend in low positives. This highlights the greater sensitivity of the S3 protocol in detecting lower spore concentrations.

**Figure 4:**
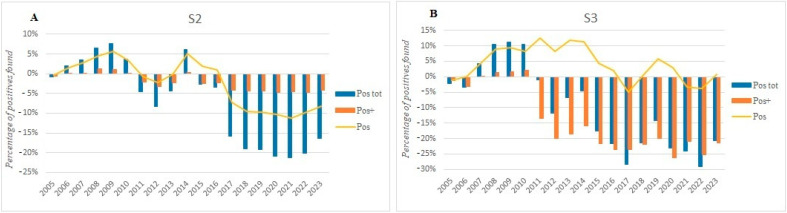
The 20-year annual trend in positive samples from S2 (**A**) and S3 (**B**) analyses. Total (Pos tot), weak (Pos), and strong (Pos+) positive samples are reported as blue bars, yellow lines, and orange bars, respectively.

This conclusion is further emphasized when comparing the positivity differentials of each year to 2004 (Figure 4). All the positive samples identified through S2 follow relatively consistent trends (Figure 4A). In contrast, S3 reveals more pronounced differences across the Pos tot, Pos, and Pos+, with the Pos+ having a dominant influence on the Pos tot (Figure 4B). Notably, between 2011 and 2015, an increase in the Pos seemed to offset the drop in Pos+. It is likely that, during this period, dairy companies began implementing strategies to combat *Clostridium* contamination. While these actions appeared to target the strong positives (Pos+), the rise in weaker positives (Pos) suggests that these initial countermeasures were not fully effective in eradicating contamination but may have reduced its severity.

The trend towards a significant decrease in spore levels could provide valuable information for dairies, especially those increasingly inclined to replace silage with more environmentally friendly feeds, such as hay from stable meadows or other types of fodder. This shift could eventually reduce the need for lysozyme and promote the introduction of lactic flora, which can naturally inhibit the germination of the few remaining spores [49].

Seasonal patterns further highlight the differences between the two protocols.

While S2 showed no significant seasonal changes, S3 revealed a noticeable rise in the Pos tot, Pos, and Pos+ during the July–September period, with occasional annual peaks in October–December (Figure 3B,D,F). This suggests that S3 is more responsive to the environmental changes that affect spore contamination.

For the milk samples processed through the S2 protocol, there was no consistent seasonal trend (Figure 3A,C,D), but deviations from the mean occurred in specific years, likely due to climatic factors such as humidity and elevated temperatures. Interestingly, the raw cow milk tested with the S3 protocol during the summer showed a significant increase in Clostridium spores, probably due to the elevated temperatures and the cows’ physiological changes. This is supported by the studies demonstrating that environmental factors can significantly influence microbial levels [16]. Research showing a correlation between increased *Clostridium* spores and warmer climatic conditions supports the idea that temperature, rather than fixed seasonal variability, may play a major role in fluctuating contamination levels [50].

Furthermore, the changes observed in positivity rates during the warmer seasons may be attributed not only to the improved survival and adaptation of *Clostridium* spores in higher temperatures but also to the metabolic and physical changes in cows during the summer [51]. Additionally, it is essential to consider how evolving climatic conditions—both on farms and in dairies—can impact contamination levels through factors such as dust, humidity, and the type of bedding used.

## 5. Conclusions

Butyric acid bacteria are the main culprits of various types of defects in cheeses. Our a-posteriori analysis aims to describe how their prevalence in raw cow milk has changed over a 20-year period. The S2 and S3 protocols of the MPN method, applied to the bovine samples delivered to IZSLER from its area of competence, have shown reductions in *Clostridium* contamination in raw milk from 2004 to 2023. This reflects the positive impact of hygiene and animal welfare practices. While both methods demonstrate overall improvements, the S3 protocol, with its higher sensitivity, is more effective at detecting low spore concentrations, making it especially suitable for the targeted monitoring of milk destined for cheese-making. Finally, climatic factors, such as humidity and environmental dust, significantly influence contamination, underscoring the importance of regular monitoring.

In conclusion, our results highlight the need to choose appropriate testing protocols that consider both regulatory standards and environmental factors. This ensures the effective monitoring of barn hygiene conditions and quality control, providing clues to ensuring the welfare of the sampled animals and the quality of the resulting dairy products.

## Figures and Tables

**Table 1:** Bovine milk samples submitted for Clostridium spore analysis (2004–2023). The table summarizes all bovine milk samples delivered to IZSLER by private owners between 2004 and 2023 to assess the presence or absence of *Clostridium* spores through S2 (A) and S3 (B) methods. Samples not relevant to this study, such as milk from non-bovine species or milk that had undergone processes affecting spore survival or germination were excluded.

S2_2004–2023 (A)			
Year	n° Samples Received	n° Samples Excluded	n° Samples Included
2023	42,880	39	42,841
2022	45,023	57	44,966
2021	47,003	40	46,963
2020	41,634	30	41,604
2019	50,937	27	50,910
2018	48,982	40	48,942
2017	49,338	52	49,286
2016	48,872	28	48,844
2015	49,693	6	49,687
2014	51,170	32	51,138
2013	52,250	50	52,200
2012	53,325	73	53,252
2011	52,178	146	52,032
2010	49,768	109	49,659
2009	49,996	72	49,924
2008	49,797	65	49,732
2007	52,993	208	52,785
2006	61,264	252	61,012
2005	59,562	181	59,381
2004	62,563	115	62,448
**S3_2004–2023 (B)**			
**Year**	**n° Samples Received**	**n° Samples Excluded**	**n° Samples Included**
2023	3020	97	2923
2022	2326	0	2326
2021	2892	1	2891
2020	3035	20	3015
2019	4671	355	4316
2018	5131	380	4751
2017	5824	410	5414
2016	3021	226	2795
2015	2203	23	2180
2014	2116	20	1996
2013	1744	13	1731
2012	2019	1	2018
2011	2031	23	2008
2010	2244	10	2234
2009	2228	3	2223
2008	1998	5	1993
2007	2988	6	2982
2006	3599	19	3580
2005	3226	2	3224
2004	2875	1	2874

**Table 2:** Territorial distribution of raw bovine milk samples and analysis with the S2 (A) and S3 (B) protocols. The provinces included are BS (Brescia), BG (Bergamo), CR (Cremona), LO (Lodi), MI (Milano), MN (Mantua), VR (Verona), SO (Sondrio), PC (Piacenza), LC (Lecco), and PV (Pavia) with the right-hand column displaying the prevalence percentage that the provinces considered cover.

S2_2004–2023 (A)			
Year	Sampling Provinces	% Samples by Origin	Year
2023	BG, BS, CR, LO, MI, MN, VR	97.90%	2023
2022	BG, BS, CR, LO, MI, MN, VR	97.87%	2022
2021	BG, BS, CR, LO, MI, MN, VR	97.63%	2021
2020	BG, BS, CR, LO, MI, MN, VR	97.76%	2020
2019	BG, BS, CR, LO, MI, MN, VR	98.21%	2019
2018	BG, BS, CR, LO, MI, MN, VR	97.27%	2018
2017	BG, BS, CR, LO, MI, MN, VR	97.35%	2017
2016	BG, BS, CR, LO, MI, MN, VR	97.46%	2016
2015	BG, BS, CR, MN, VR	97.55%	2015
2014	BG, BS, CR, MN, VR	97.56%	2014
2013	BG, BS, CR, MN, VR	97.86%	2013
2012	BG, BS, CR, MN, VR	97.75%	2012
2011	BG, BS, CR, MN, VR	97.77%	2011
2010	BG, BS, CR, MN, VR	97.52%	2010
2009	BG, BS, CR, MN, VR	97.54%	2009
2008	BG, BS, CR, MN, VR	96.96%	2008
2007	BG, BS, CR, MN, VR	96.12%	2007
2006	BG, BS, CR, LO, MI, MN, VR	97.10%	2006
2005	BG, BS, CR, MN, VR	98.16%	2005
2004	BG, BS, CR, MN, SO, VR	98.16%	2004
**S3_2004–2023 (B)**			
**Year**	**Sampling Provinces**	**% Samples by Origin**	
2023	BG, BS, PC	97.43%	
2022	BG, BS,	96.82%	
2021	BG, BS, MN	97.23%	
2020	BG, BS, CR	96.62%	
2019	BG, BS, LC, MI, MN	97.38%	
2018	BG, BS, LC, MI	97.20%	
2017	BG, BS, LC, MI	97.8%	
2016	BG, BS, LC, MI, MN	97.46%	
2015	BG, BS	96.93%	
2014	BG, BS, CR	96.40%	
2013	BG, BS, CR, MN	95.96%	
2012	BG, BS, MN	95.14%	
2011	BG, BS, MN, SO	97.86%	
2010	BG, BS, MN, SO	96.96%	
2009	BG, BS, SO	96.85%	
2008	BG, BS, CR, SO	98.60%	
2007	BG, BS, CR, SO	98.56%	
2006	BG, BS, CR, SO	98.16%	
2005	BG, BS, CR, SO	97.08%	
2004	BG, BS, CR, PV	96.48%	

**Table 3:** Clostridium-positive samples following S2 (A) and S3 (B) analyses. The number of positives (Ps) for each dilution of the sample, MPN values (spore/L), and 95% confidence intervals are reported in A and B for samples processed with the S2 and S3 protocols, respectively. The S2 protocol includes two sample dilutions (1:10 and 1:100), while the S3 protocol also considers an undiluted sample.

S2				
P		MPN (Spore/L)		Confidence Intervals (Lower-Upper)
1 mL	0.1 mL			
0	0	<308		n.s.
0	1	308		66–1438
1	0	363		78–1697
1	1	742		159–3470
1	2	1140		244–5327
2	0	966		207–4514
2	1	1508		323–7047
2	2	2099		449–9814
3	0	3015		645–14,094
3	1	4924		1053–23,015
3	2	8704		1862–40,685
3	3	>11,000		n.s.
**S3**				
**P**			**MPN** **(Spore/L)**	**Confidence Intervals (Lower-Upper)**
**10 mL**	**1 mL**	**0.1 mL**		
0	0	0	<31	0–110
0	1	0	31	4–230
1	0	0	36	5–270
1	0	1	72	17–300
1	1	0	74	18–310
1	2	0	110	35–370
2	0	0	92	21–400
2	0	1	140	42–490
2	1	0	150	43–500
2	1	1	200	70–600
2	2	0	210	71–620
3	0	0	230	55–970
3	0	1	390	93–1600
3	1	0	430	95–1900
3	1	1	750	190–3000
3	1	2	1200	360–3700
3	2	0	930	220–4000
3	2	1	1500	440–5100
3	2	2	2200	720–6400
3	3	0	2400	560–10,000
3	3	1	4600	960–22,000
3	3	2	11,000	2500–49,000
3	3	3	>11,000	3600–n.s.

## Data Availability

The original contributions presented in the study are included in the article and Supplementary Material, further inquiries can be directed to the corresponding author.

## Supplementary Materials

The following supporting information can be downloaded at: https://www.mdpi.com/article/10.3390/foods13223638/s1, Table S1: Positivity differential between 2004 and the comparison years for total positives (Pos tot), weak positives (Pos) and strong positives (Pos+). Asterisks (*) in black and red indicate a decrease and increase in positives, respectively, Figure S1: 20-year annual relative frequency of positive samples of the S2 (A) and S3 (B) analyses. Negative, weak positive (Pos) and strong positive (Pos+) samples are shown as green line, yellow bars and red bars respectively.
