# Supplementary material for: Spore-Forming Clostridia in Raw Cow Milk from Northern Italy: A Trend Analysis over the Past 20 Years
Source: Foods. 2024 Nov 14;13(22):3638. doi: 10.3390/foods13223638 (PMC11593484; doi:10.3390/foods13223638)

Table S1. Positivity differential between 2004 and the comparison years for total positives (Pos tot), weak positives (Pos) and strong positives (Pos+). Asterisks (*) in black and red indicate a decrease and increase in positives, respectively.

S2	ΔPos tot	ΔPos	ΔPos+
2004-2005	-0,88%	-0,39%	-0,63%
2004-2006	1,92%	1,44%	0,08%
2004-2007	3,46%	2,88%	0,02%
2004-2008	6,45%	4,57%	1,23%
2004-2009	7,60%	5,68%	0,99%
2004-2010	3,70%	3,31%	0,10%
2004-2011	-4,50%	-0,82%	-2,19%*
2004-2012	-8,29%	-2,29%	-3,19%*
2004-2013	-4,45%	-0,17%	-2,28%*
2004-2014	6,08%	5,06%	0,26%
2004-2015	-2,74%	1,83%	-2,51%*
2004-2016	-3,37%	0,97%	-2,32%*
2004-2017	-15,89%*	-7,25%*	-4,11%*
2004-2018	-18,95%*	-9,62%*	-4,38%*
2004-2019	-19,22%*	-9,65%*	-4,43%*
2004-2020	-20,82%*	-10,39%*	-4,73%*
2004-2021	-21,21%*	-11,19%*	-4,59%*
2004-2022	-20,23%*	-9,72%*	-4,74%*
2004-2023	-16,48%*	-8,33%*	-4,19%*

S3	ΔPos tot	ΔPos	ΔPos+
2004-2005	-2,27%	-1,10%	-1,17%
2004-2006	-3,34%	-0,05%	-3,29%
2004-2007	4,34%	4,33%	0,10%
2004-2008	10,46%	9,01%*	1,45%
2004-2009	11,12%	9,47%*	1,66%
2004-2010	10,44%	8,25%	2,19%
2004-2011	-1,04%	12,49%*	-13,53%*
2004-2012	-11,87%	8,11%	-19,99%*
2004-2013	-6,84%	11,74%*	-18,58%*
2004-2014	-4,65%	11,30%*	-15,95%*
2004-2015	-17,47%*	4,28%	-21,75%*
2004-2016	-21,63%*	1,92%	-23,55%*
2004-2017	-28,43%*	-4,95%	-23,48%*
2004-2018	-21,43%*	0,54%	-21,97%*
2004-2019	-14,29%*	5,75%	-20,04%*
2004-2020	-23,14%*	3,00%	-26,14%*
2004-2021	-24,17%*	-3,31%	-20,86%*
2004-2022	-29,04%*	-3,69%	-25,35%*
2004-2023	-20,75%*	0,74%	-21,49%*

Figure S1: 20-year annual relative frequency of positive samples of the S2 (A) and S3 (B) analyses. Negative, weak positive (Pos) and strong positive (Pos+) samples are shown as green line, yellow bars and red bars respectively.

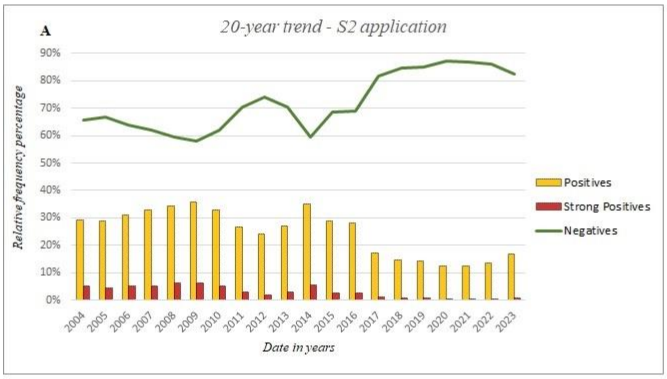

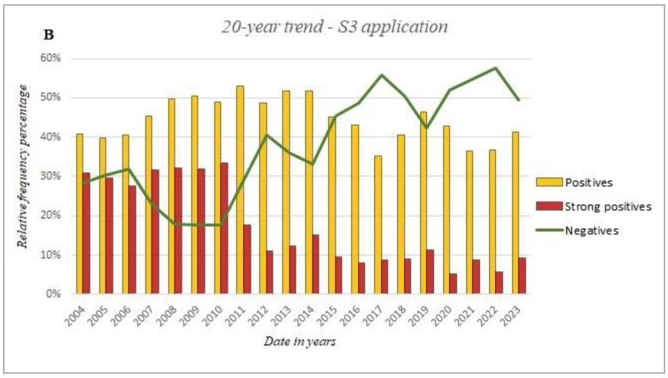

Supplement: Supplementary file 1 [file foods-13-03638-s001.zip › foods-3291212-supplementary.pdf]
